# Negotiating the plastics treaty to protect health and the environment

**DOI:** 10.2471/BLT.25.294144

**Published:** 2025-07-01

**Authors:** Nicholas Chartres, Quinn Grundy, Fiona A Miller, Björn Beeler, Tracey J Woodruff

**Affiliations:** a School of Pharmacy, Faculty of Medicine & Health, The University of Sydney, A15, Science Road, Camperdown, Sydney, NSW, 2050, Australia.; b Faculty of Nursing, University of Toronto, Toronto, Canada.; c Collaborative Centre for Climate, Health and Sustainable Care, University of Toronto, Toronto, Canada.; d International Pollutants Elimination Network (IPEN), Göteborg, Sweden.; e Program on Reproductive Health and the Environment, Department of Obstetrics, Gynecology and Reproductive Sciences, University of California, San Francisco, United States of America.

In March 2022, United Nations Member States adopted resolution EA.5/Res.4 *End plastic pollution: towards an international legally binding instrument* to negotiate an international, legally binding treaty to end plastic pollution. The resolution highlights the need to prevent plastic pollution and its related risks to human health.^1^ Yet, despite the importance of health in driving efforts to manage plastic pollution, the current proposed treaty text has major gaps that put human health at risk from hazardous chemicals and plastics. For a meaningful treaty, health considerations must figure more prominently.

The World Health Organization (WHO) estimates that approximately one quarter of all global deaths are attributable to environmental harm including chemicals, pollution and waste, disproportionately affecting low- and middle-income countries.^2^ Alarmingly, plastic production is predicted to increase by 300% by 2060.^3^

Plastic production is a key driver of unhealthy environments, with negative environmental and human health impacts at every stage of the plastics life cycle, from production to use, recycling and disposal, including littering, incineration and open burning of plastics. During the World Health Assembly (WHA) in 2023, 194 Member States raised the issue and Resolution WHA/76 was adopted to increase efforts to address pollution from chemicals and plastics, including through the Plastics Treaty process.^4^

Plastics are made of more than 16 000 chemicals, mostly derived from oil and gas.^5^ Over 4200 are known to be hazardous, while the toxicity of the majority of the remaining is unknown.^5^ These hazardous chemicals include per and poly fluoroalkyl substances (forever chemicals), phthalates (the everywhere and everyone chemicals), bisphenols and other endocrine-disrupting chemicals, which can interfere with our bodies’ natural hormone systems, leading to harmful health effects.^6^ Exposure to these and many other chemicals used in plastics has been identified to increase the risk of multiple chronic diseases, including cancer, neurodevelopmental harm and infertility.^6^

Furthermore, growing evidence points to health concerns from microplastics and nanoplastics, formed when plastics break down into small particles or are intentionally added to consumer products.^7^ Microplastics may increase the risk of respiratory, reproductive and gastrointestinal harm, with potential links to lung and colon cancer.^7^

Paradoxically, health care has become highly dependent on plastic products. The transition in health care from reusable to single-use plastic devices^8^ accelerated during the coronavirus disease 2019 pandemic, when the health sector excessively procured single-use products, with approximately half of these unused and wasted with limited evidence of benefit from the shift.^9^^,^^10^ A plastics treaty should incentivize the health sector to promote environmental sustainability and innovations for safer materials.

Most countries participating in the treaty favour addressing plastic pollution at every stage of the plastic lifecycle, including setting limits on plastics production.^11^ However, progress is being stifled by a coalition of oil- and gas-producing countries and industry groups representing fossil fuel, chemical and plastics companies, which advocate for a treaty focused on waste management and recycling, an approach that has exacerbated, not mitigated, the harms of plastics.^12^ These countries and companies also seek to limit discussions to plastic products, which detracts from a focus on regulating toxic chemicals in plastic as a material. An approach regulating plastic as a material would more effectively address all plastic, from production throughout the life cycle.

Therefore, when treaty talks continue in August 2025, we recommend that delegates address WHA concerns on the human and environmental health impacts of plastics and create a treaty that: (i) protects health and the environment as core treaty objectives; (ii) mandates consideration of health risks and impacts in all relevant treaty obligations and decisions; (iii) focuses on capping and reducing plastic production and incentivizing alternatives; (iv) ends production and use of toxic chemicals in all plastics and ensures safe, toxics-free alternatives while preventing substitution with similar hazardous chemicals; (v) removes toxic releases and emissions at all stages of the lifecycle of plastics, including banning recycling of plastics that contain toxic chemicals; (vi) requires reporting, transparency and accountability on plastic production and wastes, imports and exports (including their associated chemicals); (vii) utilizes all financing mechanisms to implement the treaty, including via extended producer responsibility and the polluter pays principle; and (viii) rejects blanket exemptions, including plastics for health care, while ensuring essential medicines and health products remain accessible and affordable to those who need them.

These steps align with WHO’s position on the treaty^11^ and are essential to fulfilling its mandate to protect health and the environment from toxic plastics.
